# Electro-Oxidation and Electro-Fenton Degradation of PFASs Using a Grid-Shaped Ti_4_O_7_ Magnéli-Phase Anode: Effect of Concentration and Evidence of Defluorination

**DOI:** 10.3390/ma19081659

**Published:** 2026-04-21

**Authors:** Sinda Daghfous, Elissa Makhoul, Eddy Petit, Geoffroy Lesage, Mikhael Bechelany, Nizar Bellakhal, Marc Cretin

**Affiliations:** 1 Institut Européen des Membranes-IEM (UMR 5635), CNRS, ENSCM, University of Montpellier, 34095 Montpellier, France; sinda.daghfous@umontpellier.fr (S.D.); elissa.makhoul@umontpellier.fr (E.M.); geoffroy.lesage@umontpellier.fr (G.L.); mikhael.bechelany@umontpellier.fr (M.B.); 2 EcoChimie Laboratory, National Institute of Applied Sciences and Technology, University of Carthage, Centre Urbain Nord, BP 676, Tunis Cedex 1080, Tunisia; nizar.bellakhal@insat.ucar.tn; 3 Plateforme d’Analyses et de Caractérisations PAC Chimie Balard (UAR 2041), CNRS, ENSCM, University of Montpellier, 34095 Montpellier, France; eddy.petit@umontpellier.fr

**Keywords:** PFAS, PFOA, PFOS, electro-oxidation, electro-Fenton, Ti_4_O_7_, Magnéli phase, defluorination, advanced oxidation processes

## Abstract

**Highlights:**

**What are the main findings?**

- Grid-shaped Ti_4_O_7_ enables efficient PFOA/PFOS electro-oxidation.
- EO-EF mainly accelerates kinetics, with a stronger benefit for PFOA.
- At 0.2 ppm (EO-EF), fluoride release indicates partial C-F bond cleavage.

**What are the implications of the main findings?**

- EO-EF mitigates organic-matter inhibition compared with EO alone.
- Ti_4_O_7_ anodes are promising for dilute PFAS treatment at moderate energy demand levels.

**Abstract:**

The persistence of per- and polyfluoroalkyl substances (PFASs) in aquatic environments requires efficient and sustainable treatment technologies. In this study, the electrochemical degradation of perfluorooctanoic acid (PFOA) and perfluorooctanesulfonic acid (PFOS) was investigated using a grid-shaped Ti_4_O_7_ Magnéli-phase anode under electro-oxidation (EO) and electro-oxidation coupled with electro-Fenton (EO-EF) conditions. Structural characterization confirmed the predominance of Ti_4_O_7_ in the electrode material. At an initial concentration of 2 ppm, PFOS was rapidly and almost completely removed under both EO and EO-EF, whereas PFOA exhibited slower degradation kinetics, identifying it as the kinetically limiting compound. Coupling EO with electro-Fenton mainly enhanced the degradation kinetics, particularly for PFOA, while final removal efficiencies remained comparable. The influence of initial concentration was further examined, showing that lowering the PFOA concentration to 0.2 ppm, representative of environmentally relevant levels, enabled nearly complete removal within 300 min. Fluoride ion monitoring under optimized EO-EF conditions confirmed partial defluorination, demonstrating that PFOA removal is accompanied by C-F bond cleavage. These findings highlight the respective roles of EO and EO-EF processes and support the potential of Ti_4_O_7_-based anodes for energy-competitive PFAS remediation.

## 1. Introduction

Per- and polyfluoroalkylated substances (PFASs), including perfluorooctanoic acid (PFOA) and perfluorooctanesulfonic acid (PFOS), are synthetic compounds widely used in industrial and domestic applications such as fire-fighting foams, textiles, food packaging, and medical devices. Their extensive use is attributed to their remarkable chemical and thermal stability [1,2]. Structurally, PFASs consist of a hydrophobic fluorinated chain and a hydrophilic head group—carboxylic for PFOA and sulfonic for PFOS—which strongly influence their behavior in aqueous media and their reactivity during electrochemical treatment [3,4]. In recent years, PFASs have been classified among the most concerning persistent organic contaminants due to their low biodegradability, environmental mobility, and bioaccumulative potential [5].

The treatment of PFASs remains a major challenge because of the exceptional strength of the carbon–fluorine bond, which makes them resistant to conventional degradation methods such as adsorption, membrane filtration, and chemical oxidation [6]. Consequently, recent research has focused on advanced technologies including photocatalysis, plasma treatment, and advanced electrochemical oxidation processes (EAOPs), which have emerged as promising alternatives [7,8,9]. Among them, electro-oxidation stands out due to its ability to generate highly reactive hydroxyl radicals (•OH) directly on the anode surface, capable of mineralizing a wide variety of persistent pollutants without requiring additional reagents [10].

The performance of electro-oxidation largely depends on the type of anode material. “Active” anodes (Pt, IrO_2_, RuO_2_) exhibit limited mineralization efficiency due to their preference for the oxygen evolution reaction [11]. In contrast, “non-active” an-odes such as boron-doped diamond (BDD) and sub-stoichiometric TiO_2_-based Magnéli phases promote the generation of adsorbed hydroxyl radicals, enabling higher oxidation efficiency [12,13,14].

Among Magnéli phases (Ti_n_O_2n−1_, 3 ≤ n ≤ 10), Ti_4_O_7_ has attracted great attention for its high electrical conductivity, chemical stability in acidic media, and long-term durability (Wang et al. [15], Tan et al. [16]). This material has proven effective for degrading various organic pollutants including antibiotics [17,18], and phenolic com-pounds [19]. In PFAS degradation, several studies have highlighted the high efficiency of Ti_4_O_7_-based anodes for PFOA and PFOS removal via electro-oxidation [20].

Ti_4_O_7_ is typically synthesized by the thermal reduction of TiO_2_ in reducing atmospheres (e.g., H_2_/N_2_) at 600–1000 °C [21]. Plasma-assisted carbothermal reduction and double annealing (oxygen then hydrogen) are alternative routes that ensure stable crystal-line structures while preserving the morphology of TiO_2_ nanotubes [22]. However, achieving pure crystalline phases remains difficult because synthesis is highly sensitive to parameters such as temperature, atmosphere, and duration, often resulting in multiphase materials. Although such coexistence can lead to synergistic effects, it complicates the attribution of electrochemical activity to a specific phase. Therefore, rigorous characterization—using XRD, Raman spectroscopy, and electron microscopy—is essential to validate phase composition and performance [23,24].

The chemical structure of the PFAS used also influences its electrochemical behavior. PFOS typically exhibits stronger adsorption on Ti_4_O_7_-based anodes than PFOA, enhancing its degradation rate. This is attributed to its higher acidity (pKa ≈ −3.27 for PFOS vs. 0.74–2.58 for PFOA), which strengthens electrostatic interactions with the anode surface [4]. Density functional theory (DFT) simulations confirm that PFOS displays a higher adsorption energy on Ti_4_O_7_, reflecting stronger interaction and enhanced oxidation efficiency.

To further improve degradation efficiency, coupling electro-oxidation with the electro-Fenton process has been proposed. This hybrid system generates H_2_O_2_ at the cathode through oxygen reduction; H_2_O_2_ then reacts with Fe^2+^, added to the solution as a catalyst, to form additional hydroxyl radicals within the solution, thereby increasing radical density both at the electrode surface and in the bulk phase [25,26,27,28]. Such coupling is particularly advantageous in complex matrices where pollutant interactions and organic matter can limit anodic oxidation alone.

In this study, we evaluate the performance of a Magnéli-phase Ti_4_O_7_ anode for the electro-chemical degradation of PFOA and PFOS, comparing electro-oxidation alone with its coupling to the electro-Fenton process. The Ti_4_O_7_ anode demonstrated low specific energy consumption (0.018–0.038 kWh.m^−3^ at 2 ppm PFOA), highlighting the potential of Magnéli-phase electrodes to achieve efficient PFAS degradation with moderate energy demand.

These results confirm the relevance of Ti_4_O_7_-based systems for the electrochemical treatment of PFAS-contaminated waters and support further investigation of their performance under varying concentrations and matrix conditions.

Experiments conducted in the presence of organic matter (OM) allowed us to evaluate the influence of OM and to compare its impact under EO and EO-EF conditions.

Therefore, this study aims to assess the electrochemical performance, energy efficiency, and degradation selectivity of an optimized Ti_4_O_7_ Magnéli-phase anode for the treatment of PFOA and PFOS under electro-oxidation and coupled electro-Fenton conditions. The findings are expected to bridge the gap between laboratory-scale and practical PFAS remediation, offering insights for the development of energy-efficient and durable electrochemical materials.

## 2. Materials and Methods

### 2.1. Material

Carbon felt was bought from Alfa Aesar. Perfluorooctanoic acid (PFOA, 95% CAS number 335-67-1), perfluorooctanesulfonic acid (PFOS, ~40% CAS number 1763-23-1), ferrous sulfate heptahydrate (FeSO_4_·7H_2_O CAS number 7782-63-0), anhydrous sodium sulfate (Na_2_SO_4_, 95% CAS number 7757-82-6), and sulfuric acid (CAS number 7664-93-9), were supplied by Sigma-Aldrich (Saint Quentin Fallavier, France). Solutions were made using Milli-Q water (Milli-Pore Quantum^®^ TEX resin system, Merck S. A., Lyon, France).

### 2.2. Anode Synthesis

A mixture of TiO_2_ (Altichem, >98%, Cergy Pontoise, France) and coke (Coke de Brai AO151203, Altichem, 98% C, Cergy Pontoise, France) was introduced into a Heroult furnace, where an electric arc was generated between two graphite electrodes. The arc melted the mixture, which was poured into a graphite mould to form an ingot. The obtained ingot was jaw-crushed, milled, and sieved to produce a powder used as feedstock for plasma spraying.

The plasma coating was deposited onto a titanium substrate (4 × 6 cm) using a ProPlasma STD torch equipped with a tungsten cathode and a copper annular anode from the company Saint Gobain Coating Solution, Cavaillon, France. A mixture of argon and hydrogen (19.6% H_2_) was introduced between the electrodes, and a direct current potential was applied to generate an electric arc (38 kW). The resulting plasma plume reached an inner temperature range of 10,000–15,000 °C.

The powder was injected into the plasma plume using argon as carrier gas (30 g·min^−1^), with an injector diameter of 1.8 mm and an injection angle of +10°. The particles were melted and accelerated toward the titanium substrate, which had been previously sandblasted to enhance surface roughness and coating adhesion. Upon impact, the molten particles formed splats that rapidly solidified on contact with the cold substrate. A homogeneous and continuous lamellar coating was obtained by moving the torch relative to the substrate at a linear velocity of 800 mm·s^−1^ with a step size of 2 mm.

### 2.3. Physico-Chemical Characterization

A Hitachi S4800 scanning electron microscope (SEM) and a VHX-7000 3D optical microscope (KEYENCE, Osaka, Japan) were used to examine the surface morphology of the anode. In addition, Raman spectral data were obtained using dispersive Raman spectroscopy (HORIBA LABRAM, Lyon, France) with a 659 nm wavelength laser operating at a constant power of 20 W. Phase identification was carried out using X-ray diffraction (XRD) on a PANalytical X’Pert (Malvern Panalytical, Palaiseau, France) diffractometer with Cu Kα radiation (λ = 0.15406 nm), and diffraction patterns were recorded in the 2θ range of 20° to 60°.

### 2.4. Electrochemical Degradation Experiments

#### 2.4.1. Anodic Electro-Oxidation

The anodic electro-oxidation was conducted in a 200 mL beaker, wherein the Magnéli phase anode and the carbon felt cathode are immerged. The anode electrode is rectangular in shape, (5 cm × 6 cm × 2 mm). During the course of the treatment, the anode is immersed in the solution within the central compartment of the cell, occupying an area of approximately 30 cm^2^. The carbon felt is positioned to surround the entire beaker, with a surface area of 176 cm^2^ (22 cm × 8 cm). The distance between the carbon felt and the anode was 2.5 cm. To ensure ionic transport in the solution prepared with PFOA or PFOS, Na_2_SO_4_ (50 mM) was added. The current density was 13 mA.cm^−2^ and was generated by a DC power generator (ELC DC Power supply AL78NX from RS Components, Beauvais, France). The electro-oxidation treatment lasted 5 h.

#### 2.4.2. Anodic Electro-Oxidation Coupled with Electro-Fenton

In order to combine electro-oxidation with the electro-Fenton process, a concentration of 0.2 mM ferrous sulphate heptahydrate (FeSO_4_-7H_2_O) was added to the solution. The presence of FeSO_4_-7H_2_O is pivotal in facilitating the Fenton reaction, where in the Fe^2+^ ions generated serve as the catalyst for the decomposition of hydrogen peroxide (H_2_O_2_) into hydroxyl radicals (•OH), which is the oxidising agent. The pH of the solution was adjusted to 3 using a drop of concentrated sulphuric acid, as it has been determined that this is the optimum value for the Fenton reaction, as it promotes the stability of Fe^2+^ and the efficient formation of hydroxyl radicals (Brillas et al. [29])

To generate H_2_O_2_ in situ continuously, a flow of oxygen gas (supplied by Air Liquide, purity ≥ 99.5%) was blown into the solution throughout the experiments. The reduction of dissolved oxygen at the cathode is achieved through the reaction O_2_ + 2H^+^ + 2e^−^ → H_2_O_2_, enabling continuous generation of the oxidizing agent without the need for external addition. This apparatus ensures the creation of an optimal electrochemical environment for the advanced degradation of organic pollutants by the electro-Fenton process.

#### 2.4.3. Data Treatment

The removal rate was calculated from concentration measurements according to

$$Removal\;rate\;\left(\%\right)=(1-\frac{Ct}{C0})\times 100$$

where *C*0 is the initial concentration and *Ct* is the concentration at time *t*. The half-life time *t*_1/2_ corresponds to 50% conversion (*Ct*/*C*0 = 0.5).

### 2.5. PFOA and PFOS Analysis

PFOA concentrations (before and after electrochemical treatment) and degradation by-products were monitored by UHPLC-MS/MS (Shimadzu-8050^®^, Shimadzu, Noisel, France) equipped with a Kinetex^®^ EVO C18 column (50 mm × 2.1 mm–1.7 μm–100 Å, Phenomenex, Torrance, CA, USA). The mobile phases consisted of 0.1% formic acid in water (A) and 0.1% formic acid in acetonitrile (B). Chromatographic separation was performed at the flow rate of 0.5 mL min^−1^ with the following gradient: 0.00 min, 2% B → 0.50 min, 50% B → 3.50 min, 80% B → 4.10 min, 2% B → Stop at 5.00 min. The injection volume was 1 μL. The column temperature was maintained at 15 °C. Electrospray ionization mass spectrometry was conducted in MRM (Multiple Reaction Monitoring) in negative mode. The source parameters were set were as follows: desolvation line temperature, 200 °C; desolvation interface temperature, 450 °C; drying and heating gas flow rate, 8 L min^−1^, and nitrogen nebulization gas flow rate 2.5 L min^−1^. The limit of quantification (LOQ) was 50 ppb.

### 2.6. Fluoride Determination and Calibration Procedure

Fluoride concentrations were measured using a fluoride ion-selective electrode (ISE) connected to a Mettler-Toledo SevenDirect SD50 ion meter. Prior to sample analysis, the electrode was calibrated using NaF standards ranging from 10^−2^ to 10^−6^ mol.L^−1^(including intermediate concentrations: 10^−3^, 10^−4^, 2 × 10^−5^, 4 × 10^−5^, 6 × 10^−5^, 1 × 10^−5^, 8 × 10^−6^, 6 × 10^−6^, 4 × 10^−6^, 2 × 10^−6^, and 1 × 10^−6^ mol.L^−1^). For each standard and sample, 4.5 mL of solution was mixed with 0.5 mL of TISAB (10% *v*/*v*), and the potential was recorded after a stabilization time of 180 s (3 min). The calibration curve (Figure S1) was obtained by plotting the electrode potential (mV) as a function of log_10_([F^−^]) and was used to convert sample potentials into fluoride concentrations.

Matrix blank measurements were performed using the EO-EF electrolyte (Na_2_SO_4_ 50 mM, FeSO_4_.7H_2_O 0.2 mM, pH 3) with 10% (*v*/*v*) TISAB. Fluoride concentrations were blank-corrected by subtracting the matrix blank value. Based on duplicate blank measurements (n = 2), blank-based analytical limits were estimated (see Supplementary Materials, Section S1).

### 2.7. Impact of Organic Matter on the Degradation of PFOA

The effluent utilized as a source of organic matter (OM) is the permeate from a laboratory anaerobic membrane bioreactor (AMBR), with a useful volume of 6 L. This reactor treats a synthetic wastewater, formulated to reproduce the characteristics of low-load domestic water, according to the composition described by Sánchez et al. [30]. The permeate is filtered using 0.045 μm syringe filters and stored at a temperature of 4 °C.

An analysis of the effluent revealed a chemical oxygen demand (COD) of 35.5 milligrams per liter (mg.L^−1^)and a total organic carbon (TOC) content of 11.96 mg.L^−1^.

Its ionic composition is detailed in Table 1.

A solution containing perfluorooctanoic acid (PFOA) at a concentration of 2 milligrams per liter (mg/L) was prepared using this effluent as a matrix. The objective of this study was to assess the impact of organic matter on the electrochemical performance of the Magnéli phase-based anode. The objective of this experiment is to create a simulation of conditions that more closely resemble those found in actual environments. The primary question to be addressed is whether organic matter can compete with PFOA for oxidizing species generated or active sites on the anode surface.

The treatments were carried out under the same operating conditions as used for the experiments conducted with PFOA alone: an applied current of 0.4 A, a volume of 200 mL, room temperature (~25 °C), and moderate agitation provided by a magnetic stirrer.

Samples were collected concurrently with the reference tests, and analyses were conducted by HPLC-MS, as outlined in Section 2.5.

### 2.8. Energy Consumption Calculation

The energy consumed by each treatment method after 50% degradation of PFOA or PFOS was calculated according to Equation (1):

(1) $${\mathrm{EC}}_{1/2}\;=\;\frac{\left(I\;\times \;V\;\times \;t_{1/2}\right)}{v}$$

EC_1/2_ (kWh-m^−3^) represents the specific energy consumed to achieve a 50% reduction in the initial pollutant concentration, *I* (A) is the intensity of the current applied, *V* (V) is the applied voltage, *t*_1/2_ (h) is the time required to reach half-concentration of the pollutant, and *v* (L) is the volume of solution treated.

## 3. Results

### 3.1. Physico-Chemical Characterization of the Anode

**Figure 1:**
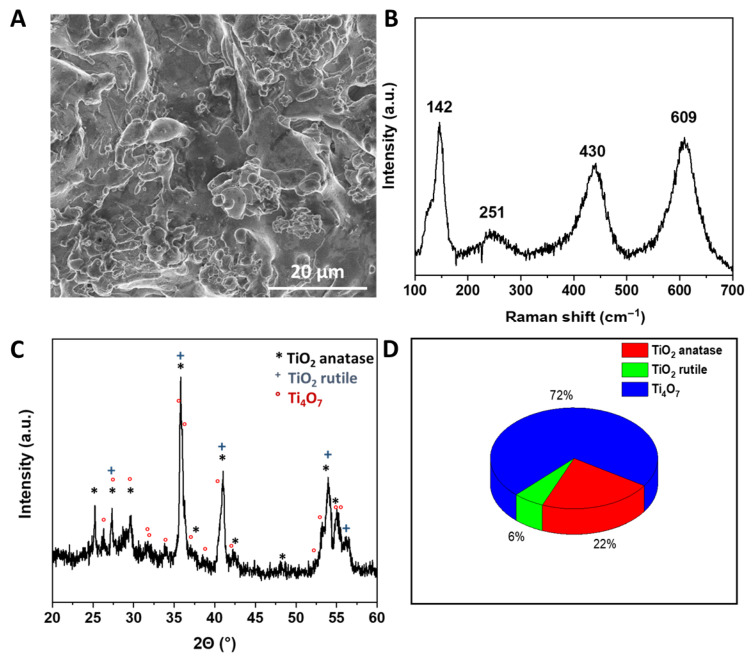
(**A**) SEM image of the surface, (**B**) Raman spectra, (**C**) XRD pattern, and (**D**) weight fractions of the crystalline phases formed in the plasma-coated TiO_x_.

The plasma-deposited anode was characterized by scanning electron microscopy (SEM), Raman spectroscopy, and X-ray diffraction (XRD). SEM observations (Figure 1A) reveal a rough and heterogeneous surface composed of agglomerated particles distributed over the titanium substrate, with typical feature sizes in the micrometer range (≈1–5 µm).

The Raman spectrum (Figure 1B) exhibits broad bands characteristic of titanium oxide phases, with contributions around ~251 cm^−1^ (B1g), ~430 cm^−1^ (Eg), and ~609 cm^−1^ (A1g), typically assigned to TiO_2_ polymorphs. A band near ~142 cm^−1^, commonly associated with Magnéli-type titanium sub-oxides, is also observed, suggesting the presence of reduced TiO_x_ phases. The broadness of Raman features is consistent with the partially disordered structure often reported for plasma-sprayed or plasma-deposited titanium sub-oxide coatings.

XRD analysis (Figure 1C) confirms the coexistence of Magnéli-type and TiO_2_ crystalline phases in the coating. The main reflections can be indexed to Ti_4_O_7_ together with TiO_2_ anatase and rutile peaks.

The main phase in the anode is Ti_4_O_7_ (72.1 ± 2.3 wt%), while secondary TiO_2_ phases (anatase and rutile) are also present, with a combined content of approximately 28.7 wt%. The formation of these secondary phases can be attributed to turbulence within the plasma plume, which likely induces local variations in temperature and oxygen partial pressure, promoting partial oxidation during deposition.

Figure 1D presents the quantitative phase composition of the anode material derived from XRD analysis. The weight fractions of the different phases were determined by applying Gaussian deconvolution to the main diffraction peaks, followed by integration of the peak areas. The relative phase content was then calculated assuming that the integrated intensities are proportional to the phase fractions.

Overall, SEM, Raman, and XRD results confirm the successful formation of a plasma-deposited titanium sub-oxide coating composed predominantly of Magnéli-type Ti_4_O_7_, with a non-negligible contribution of TiO_2_ (anatase/rutile).

### 3.2. Evaluation of the Degradation of PFOA and PFOS by Anodic Electro-Oxidation

#### Influence of PFAS Nature

The electrochemical degradation of PFOA and PFOS was investigated separately at an initial concentration of 2 ppm. Sodium sulfate (Na_2_SO_4_, 50 mM) was used as supporting electrolyte, and experiments were conducted for 5 h at a constant current density of 13 mA·cm^−2^.

**Figure 2:**
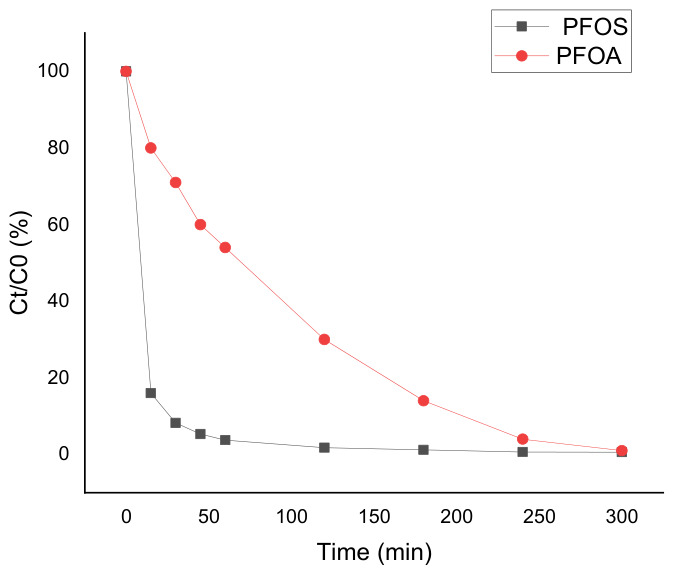
Electro-oxidation of PFOA and PFOS at an initial concentration of 2 ppm. Normalized concentration profiles (Ct/C0, %) as a function of electrolysis time for PFOA and PFOS during anodic electro-oxidation (EO) using the TiO_x_/Ti_4_O_7_ anode. Operating conditions: Na_2_SO_4_ (50 mM) as supporting electrolyte, current density J = 13 mA.cm^−2^, electrolysis time 300 min.

As shown in Figure 2, both PFOA and PFOS were efficiently removed under electro-oxidation conditions. High removal levels were reached after 300 min for both compounds. However, PFOS concentration decreased much faster than PFOA throughout the treatment. After 300 min, PFOS was almost completely removed, while PFOA required longer treatment time to reach similar removal levels. These results indicate a clear difference in degradation kinetics between the two compounds under identical operating conditions. Figure 2. Degradation of PFOA and PFOS (2 ppm) by electro-oxidation at a current density of 13 mA·cm^−2^.

### 3.3. Effect of Anodic Electro-Oxidation Coupled to Electro-Fenton Coupling on PFAS Degradation

In order to evaluate the influence of coupling electro-oxidation with the electro-Fenton process, degradation experiments were carried out under identical operating conditions in the presence of FeSO_4_·7H_2_O at pH 3, with continuous air bubbling instigated to promote in situ H_2_O_2_ generation at the cathode.

**Figure 3:**
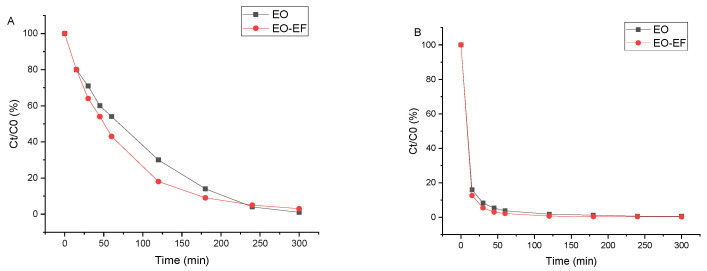
Comparison of anodic electro-oxidation (EO) and electro-oxidation coupled with electro-Fenton (EO-EF) for PFAS degradation at 2 ppm. Normalized concentration profiles (Ct/C0, %) versus electrolysis time for (**A**) PFOA and (**B**) PFOS treated by EO and EO-EF using the TiO_x_ (Magnéli-type) anode. Operating conditions: Na_2_SO_4_ (50 mM), current density J = 13 mA·cm^−2^, electrolysis time 300 min. For EO-EF: FeSO_4_·7H_2_O (0.2 mM), pH 3, and continuous air/oxygen bubbling to promote in situ H_2_O_2_ generation.

The comparative degradation profiles of PFOA and PFOS at an initial concentration of 2 ppm under EO and EO-EF conditions are shown in Figure 3.

For PFOA (Figure 3A), the EO-EF process leads to a faster decrease in concentration during the initial stage of treatment compared with EO alone. However, after 300 min, both processes reach similar removal levels.

For PFOS (Figure 3B), both EO and EO-EF lead to very rapid degradation, with nearly complete removal obtained within the first hour. Only a limited difference between EO and EO-EF is observed for this compound.

Overall, coupling EO with electro-Fenton mainly affects the degradation kinetics of PFOA, while the final removal efficiencies for both PFASs remain comparable under the investigated conditions.

The apparent pseudo-first-order kinetic constants (k) and the corresponding determination coefficients (R^2^) for the degradation of PFOA and PFOS at an initial concentration of 2 ppm under EO and EO-EF conditions are summarized in Table 2. High R^2^ values (≥0.99) indicate that the degradation profiles can be satisfactorily described by a pseudo-first-order kinetic model under all investigated conditions.

For both compounds, the coupling with electro-Fenton mainly results in an increase in the apparent reaction rate constants, confirming that EO-EF accelerates the degradation kinetics compared to EO alone. This effect is particularly noticeable for PFOA, whose apparent rate constant increases from 0.009 min^−1^ (EO) to 0.014 min^−1^ (EO-EF). For PFOS, which is already very rapidly degraded under EO conditions, the additional benefit of the Fenton coupling remains limited, with k increasing from 0.12 to 0.14 min^−1^.

These results confirm that coupling electro-oxidation with electro-Fenton leads to higher apparent reaction rates for both compounds, while maintaining high model fitting quality.

### 3.4. Influence of PFAS Concentration

**Figure 4:**
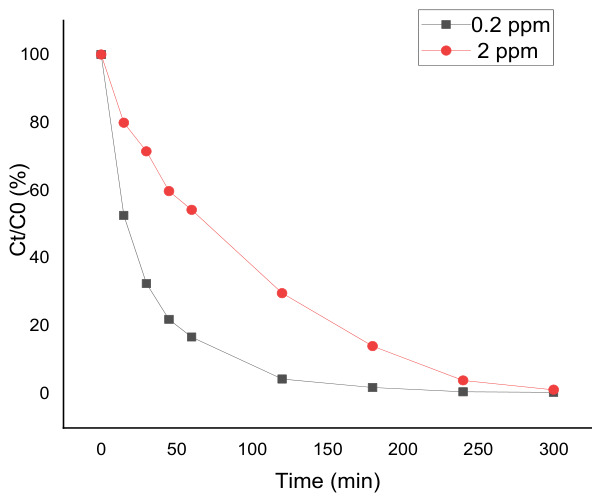
Effect of initial PFOA concentration on electro-oxidation kinetics. Normalized concentration profiles (Ct/C0, %) versus electrolysis time for PFOA treated by anodic electro-oxidation (EO) at two initial concentrations (0.2 and 2 ppm) using the TiO_x_ (Magnéli-type) anode. Operating conditions: Na_2_SO_4_ (50 mM), current density J = 13 mA·cm^−2^, electrolysis time 300 min.

The influence of the initial PFOA concentration on degradation kinetics was investigated by comparing experiments carried out at 2 ppm and 0.2 ppm under identical anodic electro-oxidation conditions. The corresponding concentration–time profiles are shown in Figure 4, and the apparent pseudo-first-order rate constants are summarized in Table 3.

A very strong effect of the initial concentration is clearly observed. At 0.2 ppm, PFOA removal is extremely fast, leading to an almost complete disappearance of the pollutant within 300 min. In contrast, at 2 ppm, although a high removal degree is also achieved, a residual fraction remains under the same operating conditions. This trend is further confirmed by the apparent kinetic constants, which increase from 0.009 min^−1^ at 2 ppm to 0.037 min^−1^ at 0.2 ppm in the present study.

This pronounced concentration effect can be explained by the relative availability of reactive oxygen species, in particular hydroxyl radicals (•OH), generated at the anode surface. At low pollutant concentration, the amount of •OH produced is largely in excess relative to the number of PFOA molecules, which increases the probability of direct radical attack and results in faster apparent degradation kinetics.

### 3.5. Defluorination Under EO-EF Conditions

Since the most favorable degradation conditions were obtained at an initial PFOA concentration of 0.2 ppm, this concentration was selected for further investigation. Apparent pseudo-first-order rate constants were determined from the slope of the linear regression of ln(Ct/C0) versus time. Similar kinetic constants were obtained for both processes, as shown in Table 4, with k values of 0.03 min^−1^ for EO and 0.027 min^−1^ for EO-EF, confirming that both treatments are highly effective at this environmentally relevant concentration.

Fluoride release was then monitored using a fluoride ion-selective electrode (ISE) under EO-EF conditions (0.2 ppm) as a proof of concept. After blank correction (EO-EF matrix + TISAB), the released fluoride concentration was 0.051 ± 0.005 mg.L^−1^, corresponding to an estimated defluorination yield of 37.3 ± 3.6% relative to the theoretical fluoride content of the initial PFOA concentration.

### 3.6. Energy Consumption

The specific energy consumption required to remove 50% of the initial PFOA concentration (EC_1/2_) was calculated as described in the Section 2.

Table 5 summarizes the apparent first-order rate constants (k), half-life values (t_1/2_), specific charge (Q), and energy consumption per cubic meter of the treated solution required to achieve 50% degradation (EC_1/2_) for both PFOA and PFOS (initial concentration 2 ppm), using anodic electro-oxidation (EO) and electro-Fenton (EO-EF) processes.

For PFOA, coupling electro-oxidation with electro-Fenton leads to a clear acceleration of degradation kinetics, as reflected by the increase in the apparent rate constant (from 0.0094 to 0.0145 min^−1^) and the decrease in half-life. At the same time, the specific energy consumption decreased from 18.45 kWh·m^−3^ in EO to 13.27 kWh·m^−3^ in EO-EF, indicating a more energy-efficient treatment despite the faster kinetics.

For PFOS, both EO and EO-EF led to very rapid degradation and comparable energy consumptions.

Table 6 presents selected literature values for energy consumption (EC) reported for electrochemical PFOA degradation under different operating conditions. Reported EC values span a wide range depending on electrode material, current density, initial concentration, reactor configuration, and the target removal level. For this reason, EC values from the literature are reported as stated by the respective authors and should be interpreted as indicative rather than as a direct benchmark.

In the present study, EC was evaluated using two complementary metrics. At an initial PFOA concentration of 2 mg.L^−1^ and for 50% conversion (t_1/2_), the energy consumption EC_1/2_ reached 18.45 kWh.m^−3^ in EO mode and 13.27 kWh.m^−3^ in EO-EF mode. In addition, the cumulative energy consumption after 300 min (EC_300_) was 75.1 kWh.m^−3^ (EO) and 83.3 kWh.m^−3^ (EO-EF), corresponding to final removals of 99% and 97%, respectively. Overall, these values fall within the range commonly reported for electrochemical PFOA treatment, while highlighting that the comparison of EC across studies strongly depends on the selected endpoint (e.g., 50% vs. near-complete removal) and operating conditions.

### 3.7. Addition of Organic Material

The influence of organic matter (OM) on PFOA degradation was investigated in order to assess the impact of matrix effects that are expected in real waters. For this purpose, experiments were carried out using the permeate of a laboratory-scale anaerobic membrane bioreactor (AnMBR) as a source of organic matter, characterized by a chemical oxygen demand (COD) of 35.5 mg.L^−1^ and a total organic carbon (TOC) of 11.96 mg L^−1^.

**Figure 5:**
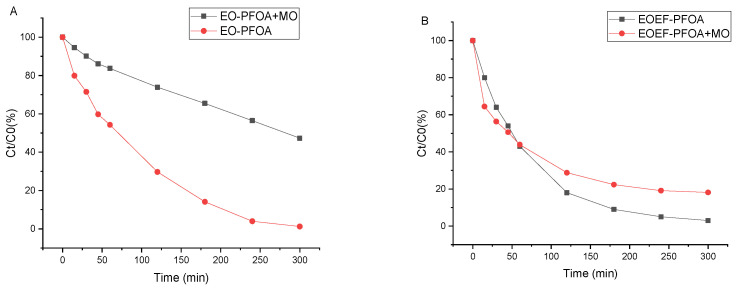
Effect of organic matter (OM) on PFOA degradation under EO and EO-EF conditions. Normalized concentration profiles (Ct/C0, %) versus electrolysis time for PFOA (initial concentration: 2 ppm) treated (**A**) by anodic electro-oxidation (EO) and (**B**) by electro-oxidation coupled with electro-Fenton (EO-EF), in the absence and presence of OM. OM was introduced using AnMBR permeate (TOC = 11.96 mg L^−1^). Operating conditions: Na_2_SO_4_ (50 mM), current density J = 13 mA.cm^−2^, electrolysis time 300 min; EO-EF conditions additionally included FeSO_4_·7H_2_O (0.2 mM), pH 3, and continuous air/oxygen bubbling.

The degradation profiles of PFOA (2 ppm) in the absence and presence of organic matter (OM) under EO and EO-EF conditions are shown in Figure 5. Under EO conditions (Figure 5A), OM markedly slows down PFOA degradation, resulting in lower removal at a given treatment time. Under EO-EF conditions (Figure 5B), the difference between the profiles obtained with and without OM is less pronounced, and both curves remain relatively close over the investigated time window.

In order to quantify these trends, apparent pseudo-first-order kinetic constants were calculated and are summarized in Table 7. Under EO conditions, the apparent rate constant decreases from 0.0094 min^−1^ for PFOA alone to 0.0025 min^−1^ in the presence of OM (−73%), confirming a strong inhibitory effect. In contrast, under EO-EF conditions, the apparent rate constant slightly increases from 0.0145 min^−1^ to 0.022 min^−1^ in the presence of OM. This unexpected trend suggests that OM may play a more complex role under EO-EF conditions, possibly acting both as a radical scavenger and as a source of secondary reactive intermediates after partial oxidation.

## 4. Discussions

The results obtained in this study provide important insights into the mechanisms governing PFAS electrochemical degradation over Ti_4_O_7_ anodes.

Under anodic electro-oxidation conditions, oxidation reactions mainly occur at the anode surface, where adsorbed hydroxyl radicals (•OH) and other reactive oxygen species are generated [27,29].

As a result, the degradation efficiency strongly depends on the interaction between the pollutant and the electrode surface.

As shown in Figure 2, both PFOA and PFOS were efficiently degraded under these conditions, reaching very high removal levels after 300 min of treatment. However, PFOS was degraded significantly faster than PFOA. This difference can be attributed to their distinct chemical structures, particularly their functional head groups. PFOS contains a sulfonic acid group, whereas PFOA contains a carboxylic acid group. Owing to its stronger acidity and higher surface affinity, PFOS exhibits stronger adsorption onto oxide-based and conductive electrode surfaces, which facilitates interfacial electron transfer and leads to faster degradation kinetics [32,33,34].

Similar trends have been reported in previous studies on the electrochemical oxidation of PFASs, where PFOS generally shows higher reactivity and faster removal than PFOA under comparable conditions [30,31,35]. This result confirms that PFOA is the more recalcitrant compound under anodic electro-oxidation conditions and should therefore be considered as the kinetically limiting case for further process optimization.

The different impact of EO-EF coupling on PFOA and PFOS can be explained by their respective reactivities toward anodic oxidation. Under the conditions described in Figure 3, hydroxyl radicals (•OH) are produced both at the anode surface and in the bulk solution through the classical Fenton reaction, which is known to enhance the oxidation of persistent organic pollutants [11,29].

For PFOS (Figure 3B), both EO and EO-EF result in very rapid and nearly complete degradation, with only a limited additional benefit brought by the Fenton coupling. This indicates that, for PFOS, anodic electro-oxidation alone is already highly efficient and that the contribution of homogeneous •OH generated by the Fenton reaction plays a secondary role.

In contrast, PFOA shows slower degradation kinetics under EO, making it the kinetically limiting compound. In this case, the presence of electro-Fenton enhances the availability of reactive oxygen species in the bulk, which accelerates the initial degradation stage. Nevertheless, since both EO and EO-EF share the same anodic oxidation mechanism, the final removal efficiency after prolonged electrolysis remains similar.

Overall, these results indicate that the main effect of EO-EF coupling in the present system is to accelerate degradation kinetics, especially for the more recalcitrant compound PFOA, rather than to fundamentally change the final removal efficiency. This observation is consistent with the fact that EO and EO-EF share the same anodic oxidation pathway, while the electro-Fenton process mainly enhances the availability of reactive species in the bulk solution [11,29,36].

The increase in apparent rate constants observed under EO-EF conditions indicates that the coupling with the electro-Fenton process mainly enhances degradation kinetics rather than significantly altering the final removal efficiency. This effect is particularly noticeable for PFOA, whose rate constant increases by approximately 36%, whereas the improvement remains more limited for PFOS (≈14%), which is already rapidly degraded under EO conditions.

Since both EO and EO-EF share the same anodic oxidation mechanism, the main role of the electro-Fenton process in the present system is to increase the availability of reactive species and thus to accelerate the initial degradation stages, rather than to fundamentally change the final removal efficiency [11,29,36]. In addition, the consistently higher apparent rate constants obtained for PFOS compared to PFOA under both processes confirm the higher reactivity of PFOS, which is in agreement with its stronger acidity and higher affinity for electrode surfaces, facilitating its preferential adsorption and faster interfacial oxidation [32,33,37,38].

The influence of initial concentration also plays an important role. At higher concentrations, the slower decay rate of the parent compound is more likely related to mass transport limitations and surface interactions between PFOA molecules and the electrode, which may reduce the effective number of active sites available for interfacial oxidation.

In addition, the accumulation of reaction intermediates at the electrode surface may influence the apparent kinetics. Such effects are commonly observed in electrochemical oxidation systems and contribute to the lower degradation rates observed at higher initial concentrations.

Such concentration-dependent kinetics have been widely reported in the literature for the electrochemical oxidation of PFASs and other persistent organic pollutants [30,32,33]. Importantly, the present results demonstrate that the Ti_4_O_7_ anode exhibits particularly high apparent kinetic performance at environmentally relevant concentrations (0.2 ppm), leading to nearly complete PFOA removal within a few hours. This highlights the strong potential of this material for the treatment of dilute PFAS-contaminated waters under realistic conditions.

Beyond parent compound removal, the occurrence of defluorination was also investigated under the most favorable degradation conditions. Although the overall removal efficiencies after 300 min were comparable, EO-EF conditions provide a higher availability of reactive species in the bulk solution, which may promote deeper oxidation pathways. For this reason, the most favorable condition (EO-EF at 0.2 ppm) was selected to investigate whether PFOA removal was accompanied by defluorination rather than only transformation into fluorinated intermediates.

Fluoride release was therefore monitored, and an increase in fluoride concentration was detected during treatment. After correction using the EO-EF matrix blank (Na_2_SO_4_ 50 mM, FeSO_4_.7H_2_O 0.2 mM, pH 3, with 10% *v*/*v* TISAB), the released fluoride concentration corresponded to an estimated defluorination yield of 37.3 ± 3.6%, calculated relative to the theoretical fluorine content of 0.2 mg.L^−1^ PFOA. This result provides proof-of-concept evidence that, under EO-EF conditions, PFOA removal can be accompanied by partial C-F bond cleavage and fluoride release. However, fluoride release alone does not demonstrate complete mineralization, as a full fluorine mass balance (including organofluorinated intermediates) would be required. In addition, because the EO-EF electrolyte contains dissolved iron at acidic pH part of the released fluoride may be complexed as Fe-F species; therefore, the ISE measurement primarily reflects free fluoride activity and the reported defluorination yield should be considered a conservative estimate.

Although defluorination was only evaluated under EO-EF conditions in the present study, these results should be regarded as a proof-of-concept demonstrating the ability of the coupled process to promote deeper transformation beyond the simple disappearance of the parent compound.

Energy consumption is another key parameter for evaluating the practical relevance of electrochemical treatments. The decrease in energy consumption under EO-EF conditions indicates a more energy-efficient treatment despite the faster kinetics.

A slight increase in cell voltage was nevertheless observed under EO-EF conditions. This may be attributed to the continuous air bubbling required for in situ H_2_O_2_ generation, which can lead to partial bubble adhesion on the electrode surface and a corresponding increase in ohmic resistance.

For PFOS, both EO and EO-EF led to very rapid degradation and comparable energy consumption, confirming that PFOS is highly susceptible to direct anodic oxidation and that the additional contribution of the electro-Fenton process remains limited in this case.

Overall, these results indicate that the benefit of EO-EF depends on the target compound and treatment objectives: while EO-EF significantly improves the kinetics for more recalcitrant species such as PFOA, electro-oxidation alone already provides an efficient and energetically favorable solution for more reactive PFASs such as PFOS [39,40].

To further place these results in context, it is important to compare the energy demand of the present system with values reported in the literature. For instance, BDD anodes typically require higher energy inputs (88–114 kWh·m^−3^) despite achieving high removal efficiencies, while hybrid Ag/Au–PAA/PAH systems may reach even higher values depending on the operating conditions.

Accordingly, to enable a clearer comparison, the energy demand of the present system is discussed using both EC_1/2_ (50% conversion) and cumulative energy consumption after 300 min (EC_300_).

In the present study, the TiO_x_ (Magnéli-type) anode exhibited moderate energy consumption. At an initial PFOA concentration of 2 mg·L^−1^ and for 50% removal, EC_1/2_ was 18.45 kWh.m^−3^ in EO mode and 13.27 kWh.m^−3^ in EO-EF mode, indicating a lower energy requirement to reach the same conversion under EO-EF conditions. In contrast, when considering the cumulative energy demand after 300 min, EC_300_ reached 75.1 kWh.m^−3^ (EO, 99% removal) and 83.3 kWh.m^−3^ (EO-EF, 97% removal), highlighting that energy comparisons depend strongly on whether EC is evaluated at a fixed conversion (e.g., 50%) or after prolonged electrolysis targeting high final removal.

These values fall within the lower range commonly reported for electrochemical PFOA treatment in the literature, highlighting the potential of Ti_4_O_7_-based anodes as energy-efficient materials for PFAS degradation. However, direct quantitative comparisons between studies remain difficult due to differences in reactor configuration, operating conditions, and target removal levels. These results nonetheless confirm that Ti_4_O_7_-based anodes can provide particularly efficient degradation at moderate current density and low concentration, combining relatively fast kinetics with reasonable energy demand.

Finally, the influence of organic matter highlights the importance of considering realistic water matrices. The degradation profiles of PFOA (2 ppm) in the absence and presence of OM under EO and EO-EF conditions are shown in Figure 5. Under EO conditions (Figure 5A), the presence of OM clearly slows down PFOA degradation, indicating a strong inhibition effect. This is reflected by a marked decrease in the apparent pseudo-first-order rate constant from k = 0.0094 min^−1^ (PFOA alone) to k = 0.0025 min^−1^ (PFOA + OM) (Table 7). This can be attributed to the competition between OM and PFOA for reactive oxygen species, in particular hydroxyl radicals (•OH), as well as to the possible partial coverage of the electrode surface by organic compounds and reaction intermediates.

Under EO-EF conditions (Figure 5B), the influence of organic matter (OM) on PFOA degradation appears less pronounced than under EO conditions alone. Although some competition between OM and PFOA for reactive species cannot be excluded, the overall degradation profiles obtained with and without OM remain relatively close. A global pseudo-first-order analysis performed over the full treatment duration yields apparent rate constants of the same order of magnitude, with k = 0.0145 min^−1^ for PFOA alone and k = 0.022 min^−1^ in the presence of OM (Table 7). However, these fitted k values should be interpreted cautiously because k is an apparent parameter derived from simplified kinetic fitting and may be sensitive to the selected fitting window and data dispersion, particularly in complex matrices. In addition, partial oxidation of OM under EO-EF may generate secondary reactive intermediates that can modify the apparent kinetics. Similar complex matrix effects have been reported for advanced oxidation processes, where coexisting organic compounds may inhibit or, in some cases, promote pollutant transformation depending on operating conditions and reaction pathways [27,29,41,42].

To better compare matrix effects under stabilized conditions, a late-stage apparent rate constant (klate) was determined by fitting only the last five sampling points (60–300 min) using a nonlinear exponential decay model (OriginPro ExpDec1, y = A_1_ exp(−t/t_1_) + y_0_, with k = 1/t_1_). This late-stage window was selected because the early part of the EO-EF profiles may correspond to a transient regime in which the oxidation environment evolves rapidly (OM competition for reactive species, progressive build-up of reactive species in the bulk solution, and formation/consumption of short-lived intermediates), whereas the late-stage region shows a more regular decay behavior. Using this approach, the late-stage apparent rate constants were klate = 0.01556 ± 6.15 × 10^−4^ min^−1^, for PFOA alone and klate = 0.01377 ± 3.65 × 10^−4^ min^−1^, in the presence of OM (Figure S2 and Table S1, Supplementary Materials; uncertainties correspond to the standard error of the fitted parameter).

Overall, these results highlight that matrix composition can influence the apparent kinetics under EO-EF and underline the importance of considering realistic water matrices when evaluating and optimizing electrochemical PFAS treatment processes.

## 5. Conclusions

This study investigated the electrochemical degradation of two representative PFASs, PFOA and PFOS, using a Ti_4_O_7_ Magnéli-phase anode under different operating conditions. The influence of PFAS nature, initial concentration (2 ppm and 0.2 ppm), coupling with the electro-Fenton process, and the presence of organic matter was systematically evaluated.

The results demonstrate that PFOS is more readily degraded than PFOA under anodic electro-oxidation conditions, confirming that PFOA should be considered the more recalcitrant compound. A pronounced concentration effect was observed, with significantly faster and nearly complete removal at environmentally relevant levels (0.2 ppm), highlighting the importance of pollutant loading on process performance.

Coupling electro-oxidation with electro-Fenton primarily enhances degradation kinetics, particularly for the more recalcitrant PFOAs, while the final removal efficiencies remain comparable to those obtained by electro-oxidation alone. Energy consumption analysis confirmed that EO-EF can improve kinetic performance without necessarily increasing energy demand, depending on the selected endpoint and target compound [27,43]. Experiments conducted in the presence of organic matter revealed a strong matrix effect under EO conditions, whereas EO-EF partially mitigated the apparent inhibition, supporting the relevance of the coupled process in more complex water matrices. Finally, fluoride measurements provided proof-of-concept evidence that, under optimized EO-EF conditions, PFOA degradation can be accompanied by fluoride release consistent with partial C-F bond cleavage, although a complete fluorine mass balance remains to be established. Overall, these findings highlight the potential of TiO_x_/Ti_4_O_7_-based anodes for PFAS treatment, particularly at low concentrations. Future work should focus on detailed degradation pathways, quantitative fluorine mass balance, and long-term electrode stability under realistic operating conditions.

## Figures and Tables

**Table 1:** Ionic composition of the anaerobic membrane bioreactor effluent used as a source of organic matter.

Component	Concentration (mg.L^−1^)
Sodium	213.66
Ammonium	8.54
Potassium	n.a.
Magnesium	11.3
Calcium	129.48
Phosphate	2.38
Nitrate	0.48
Chloride	57
Sulfate	33.08

n.a.: not available.

**Table 2:** Apparent pseudo-first-order rate constants (k) and coefficients of determination (R^2^) for PFOA and PFOS degradation (initial concentration: 2 ppm) under anodic electro-oxidation (EO) and electro-oxidation coupled with electro-Fenton (EO-EF) conditions. Operating conditions: Na_2_SO_4_ (50 mM), current density J = 13 mA.cm^−2^, electrolysis time 300 min; EO-EF additionally included FeSO_4_.7H_2_O (0.2 mM), pH 3, and air/oxygen bubbling for in situ H_2_O_2_ generation.

PFAS Concentration	PFOA—2 ppm		PFOS—2 ppm	
Process	EO	EO-EF	EO	EO-EF
First order rate constant (min^−1^)	0.009	0.014	0.12	0.14
R^2^	0.99	0.99	0.99	0.99

**Table 3:** Effect of initial PFOA concentration on apparent pseudo-first-order kinetics under anodic electro-oxidation (EO). Apparent rate constants (k) determined from ln (Ct/C0) versus time fitting for PFOA treated at two initial concentrations (2 and 0.2 ppm) using the TiO_x_ (Magnéli-type) anode. Operating conditions: Na_2_SO_4_ (50 mM), current density J = 13 mA·cm^−2^, electrolysis time 300 min.

Pollutant	Anode	Current-Density (mA cm^−2^)	Concentration (mg/L)	k (min^−1^)
PFOA	Ti_4_O_7_ (Magnéli)	13	2	0.009
			0.2	0.037

**Table 4:** Apparent pseudo-first-order rate constants (k) for PFOA degradation at 0.2 ppm under anodic electro-oxidation (EO) and electro-oxidation coupled with electro-Fenton (EO-EF) conditions. Rate constants were obtained from ln(Ct/C0) versus time fitting. Operating conditions: Na_2_SO_4_ (50 mM), current density J = 13 mA.cm^−2^, electrolysis time 300 min; EO-EF additionally included FeSO_4_.7H_2_O (0.2 mM), pH 3, and continuous air/oxygen bubbling for in situ H_2_O_2_ generation.

Pollutant	Process	Concentration (ppm)	K (min^−1^)
PFOA	EO	0.2	0.030 ± 0.003
	EO-EF	0.2	0.027 ± 0.008

**Table 5:** Energy consumption at half-conversion (EC_1/2_) for PFOA and PFOS degradation (initial concentration: 2 ppm) under anodic electro-oxidation (EO) and electro-oxidation coupled with electro-Fenton (EO-EF) conditions. EC_1/2_ (kWh.m^−3^) corresponds to the electrical energy required to reach 50% degradation (t_1/2_) Operating conditions: Na_2_SO_4_ (50 mM), current density J = 13 mA·cm^−2^, electrolysis time 300 min; EO-EF additionally included FeSO_4_.7H_2_O (0.2 mM), pH 3, and continuous air/oxygen bubbling for in situ H_2_O_2_ generation.

Pollutant	Process	I (A)	U (V)	k (min^−1^)	t_1/2_ (min)	EC_1/2_ (kWh.m^−3^)
PFOA	EO	0.4	7.5	0.0094 ± 0.0008	73.73	18.45
PFOA	EO-EF	0.4	8.4	0.0145 ± 0.0008	47.80	13.27
PFOS	EO	0.4	7.5	0.126 ± 0.011	5.50	1.38
PFOS	EO-EF	0.4	8.4	0.140 ± 0.009	4.95	1.40

**Table 6:** Selected literature values of energy consumption (EC) for electrochemical PFOA degradation, as well as EC values obtained in this study at 50% removal (EC_1/2_) and after 300 min (EC_300_).

Pollutant	Concentration	Removal Rate (%)	EC (kWh.m^−3^)	Anode	Process	Current Density (mA.cm^−2^)	Reference
PFOA	1350 ng.L^−1^	80	88–114	BDD	EO	75	Pierpaoli et al. [30]
PFOA	1 µg.L^−1^	72	164.9	Ag/Au-PAA/PAH	EO	10	Hwang et al. [31]
PFOA	2 mg.L^−1^	50	18.45	Ti_4_O_7_	EO	13	This study
PFOA	2 mg.L^−1^	50	13.27	Ti_4_O_7_	EO-EF	13	This study
PFOA	2 mg.L^−1^	99	75.1	Ti_4_O_7_	EO	13	This study
PFOA	2 mg.L^−1^	97	83.3	Ti_4_O_7_	EO-EF	13	This study

**Table 7:** Effect of organic matter (OM) on apparent kinetic constants for PFOA degradation (2 ppm) under electro-oxidation (EO) and electro-oxidation coupled with electro-Fenton (EO-EF) conditions. Operating conditions: Na_2_SO_4_ (50 mM), current density J = 13 mA·cm^−2^, electrolysis time 300 min; EO-EF additionally included FeSO_4_.·7H_2_O (0.2 mM), pH 3, and continuous air/oxygen bubbling. OM was introduced using AnMBR permeate (TOC = 11.96 mg L^−1^).

PFAS Concentration	Electrode Ti_4_O_7_			
	Electro-Oxidation		Electro-Oxidation Coupled with Electro Fenton	
	First-Order Rate Constant (min^−1^)	R^2^	First-Order Rate Constant (min^−1^)	R^2^
**PFOA—2 ppm**	0.0094	0.97	0.0145	0.99
**PFOA + OM—2 ppm**	0.0025	0.99	0.022	0.99

## Data Availability

The original contributions presented in this study are included in the article/Supplementary Material. Further inquiries can be directed to the corresponding author.

## Supplementary Materials

The following supporting information can be downloaded at: https://www.mdpi.com/article/10.3390/ma19081659/s1, Figure S1: Fluoride ISE calibration curve. Electrode potential E (mV) measured with a Mettler-Toledo SevenDirect SD50 after 180 s (3 min) stabilization. Standards: NaF, 10^−2^–10^−6^ mol·L^−1^ (in-cluding 10^−3^, 10^−4^, 2 × 10^−5^, 4 × 10^−5^, 6 × 10^−5^, 1 × 10^−5^, 8 × 10^−6^, 6 × 10^−6^, 4 × 10^−6^, 2 × 10^−6^, 1 × 10^−6^ mol·L^−1^) prepared in Milli-Q water in the EO-EF matrix (Na_2_SO_4_ 50 mM, FeSO_4_·7H_2_O 0.2 mM, pH 3). TISAB 10% (*v*/*v*) was added prior to measurement (4.5 mL standard + 0.5 mL TISAB). Plot: E vs. log_10_([F^−^]). Linear fit: E (mV) = −50.641 log_10_([F^−^]) − 130.2, R^2^ = 0.987; Figure S2. Late-stage degradation profiles used for kinetic analysis under EO-EF conditions. Normalized PFOA concentration (C/C0, %) for EO-EF treatment at 2 ppm in the absence and presence of organic matter (OM), shown for the late-stage region (last five sampling points, t = 60–300 min) used to determine klate; Table S1. Late-stage apparent pseudo-first-order rate constants under EO-EF conditions (2 ppm PFOA). Late-stage rate constants were obtained by the nonlinear exponential fitting (OriginPro ExpDec1, y = A1 exp(−t/t1) + y0, with k = 1/t1) of the last five sampling points (t = 60–300 min). Reported uncertainties correspond to the standard error (SE) of the fitted parameter.

## Abbreviations

PFAS	Per- and polyfluoroalkyl substances
PFOA	Perfluorooctanoic acid
PFOS	Perfluorooctanesulfonic acid
EO	Electro-oxidation
EO-EF	Electro-oxidation coupled with electro-Fenton
EF	Electro-Fenton
OM	Organic matter
COD	Chemical oxygen demand
TOC	Total organic carbon
AMBR	Anaerobic membrane bioreactor
ROS	Reactive oxygen species
•OH	Hydroxyl radical
Ti_4_O_7_	Titanium sub-oxide Magnéli phase
BDD	Boron-doped diamond
XRD	X-ray diffraction
SEM	Scanning electron microscopy
k	Apparent first-order rate constant
R^2^	Coefficient of determination
T_1/2_	Half-life time
EC	Energy consumption
EC_1/2_	Energy consumption at 50% removal
Q	Electric charge
J	Current density
I	Current
U	Cell voltage
