# Supplementary material for: Electro-Oxidation and Electro-Fenton Degradation of PFASs Using a Grid-Shaped Ti4O7 Magnéli-Phase Anode: Effect of Concentration and Evidence of Defluorination
Source: Materials (Basel). 2026 Apr 21;19(8):1659. doi: 10.3390/ma19081659 (PMC13117976; doi:10.3390/ma19081659)
Supplement: Supplementary file 1 [file materials-19-01659-s001.zip › materials-4224171-SI.pdf]

Section S1. Fluoride ISE calibration and analytical performance

A linear Nernstian response was obtained over the investigated range (10^{-2}–10^{-6} mol·L^{-1}). The calibration equation derived from Figure S1 was
E (mV) = –50.641 log$_{10}$([F$^-$]) – 130.2, with **R^2 = 0.987**.

To account for matrix effects, blank measurements were performed using the EO–EF matrix (Na_2SO_4 50 mM, $FeSO_4{\cdot}7H_2O$ 0.2 mM, pH 3) with TISAB at 10% (v/v). Duplicate blank potentials (166.8 and 167.5 mV; n = 2) were used to estimate blank-based analytical limits from the calibration slope (LOD = 3.3σ and LOQ = 10σ, where σ is the standard deviation of blank potentials). The estimated limits were **LOD = 1.45 × 10^{-6} mol·L^{-1} (0.0275 mg·L^{-1} as F$^-$)** and **LOQ = 1.68 × 10^{-6} mol·L^{-1} (0.0319 mg·L^{-1} as F$^-$)**.

The sample potential was measured twice (143.8 and 146.5 mV), yielding **145.2 ± 1.4 mV**, which falls within the validated linear range and corresponds to a fluoride concentration above the estimated LOQ. After correction for dilution with TISAB, the measured fluoride concentration was **0.077 ± 0.005 mg·L^{-1}**. After blank correction, the released fluoride concentration was **0.051 ± 0.005 mg·L^{-1}**, corresponding to a defluorination yield of **37.3 ± 3.6%** relative to the theoretical fluorine content of **0.2 mg·L^{-1} PFOA**.

Note: Because the EO–EF matrix contains dissolved iron at acidic pH, part of the released fluoride may be complexed as Fe–F species. Therefore, the ISE signal primarily reflects free fluoride activity and the reported fluoride release/defluorination yield should be considered conservative estimates.

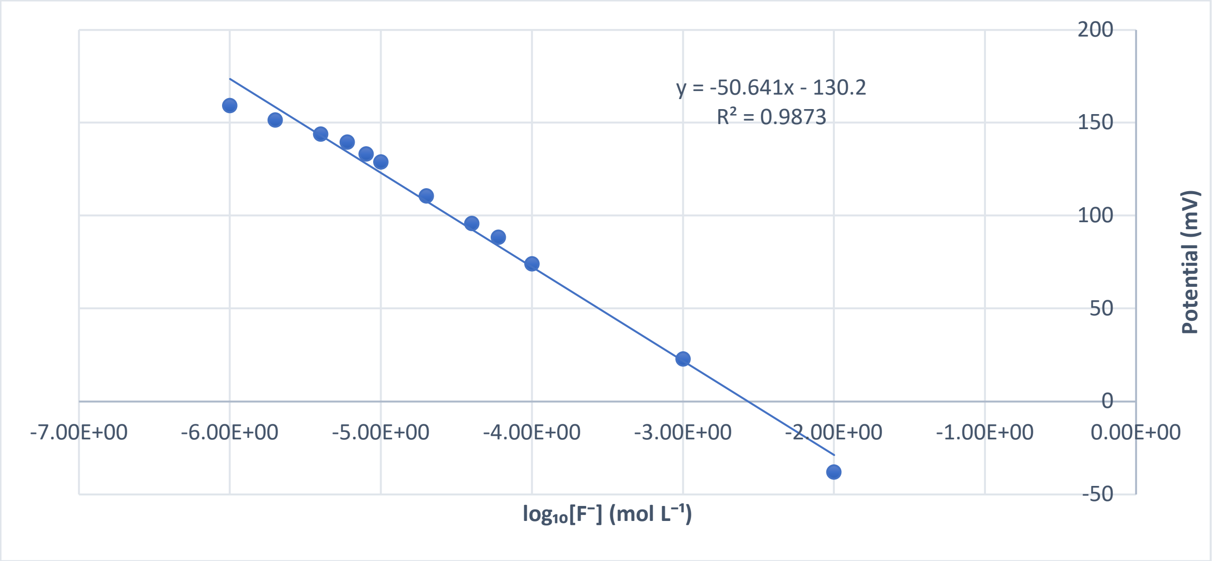

Figure S1. *Fluoride ISE calibration curve.* Electrode potential **E (mV)** measured with a Mettler-Toledo SevenDirect SD50 after **180 s (3 min)** stabilization. Standards: **NaF, 10^{-2}–10^{-6} mol·L^{-1}** (including 10^{-3}, 10^{-4}, $2{\times}10^{-5}$, $4{\times}10^{-5}$, $6{\times}10^{-5}$, $1{\times}10^{-5}$, $8{\times}10^{-6}$, $6{\times}10^{-6}$, $4{\times}10^{-6}$, $2{\times}10^{-6}$, $1{\times}10^{-6}$ mol·L^{-1}) prepared in **Milli-Q water** in the **EO–EF matrix** *(Na_2SO_4 50 mM, $FeSO_4{\cdot}7H_2O$ 0.2 mM, pH 3)*. **TISAB 10% (v/v)** was added prior to measurement *(4.5 mL standard + 0.5 mL TISAB)*. Plot: **E vs log$_{10}$([F$^-$])**. Linear fit: **E (mV) = –50.641 log$_{10}$([F$^-$]) – 130.2**, **R^2 = 0.987**.

Section S2. Supporting results for kinetic analysis in the presence/absence of organic matter (EO–EF).

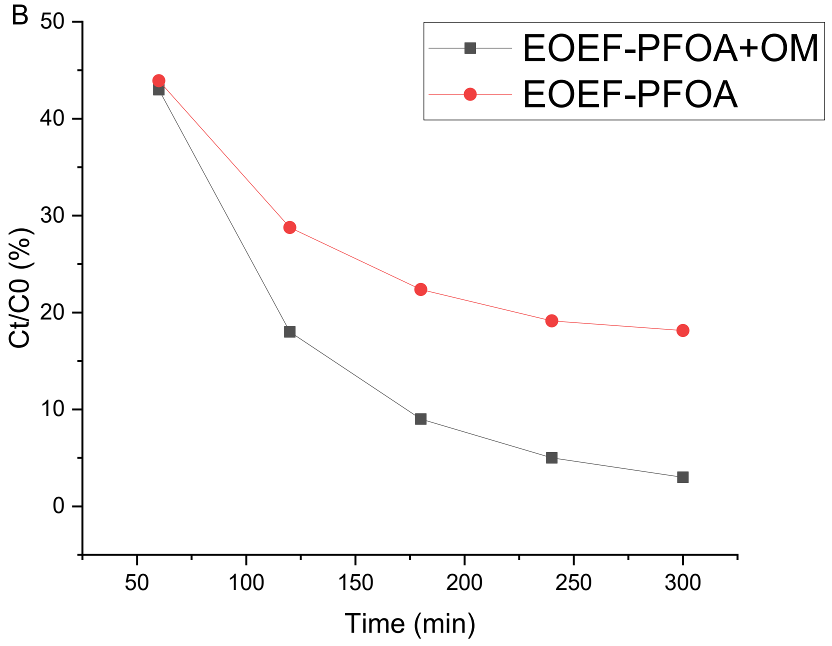

Figure S2. Late-stage degradation profiles used for kinetic analysis under EO–EF conditions. Normalized PFOA concentration (C /C0, %) for EO-EF treatment at 2 ppm in the absence and presence of organic matter (OM), shown for the late-stage region (last five sampling points, t = 60-300 min) used to determine klate.

Table S1. Late-stage apparent pseudo-first-order rate constants under EO–EF conditions (2 ppm PFOA). Late-stage rate constants were obtained by the nonlinear exponential fitting (OriginPro ExpDec1, $y = A_1 \exp(-t/t_1) + y_0$, with $k=1/t_1$) of the last five sampling points (**t = 60–300 min**). Reported uncertainties correspond to the standard error (SE) of the fitted parameter.

Condition	Time window (min)	k (min^{-1})	SE (min^{-1})
EO-EF, PFOA	60-300	0.01556	6.15×10^{-4}
EO-EF, PFOA + OM	60-300	0.01377	3.65×10^{-4}
